# Gut Microbiome Prolongs an Inhibitory Effect of Korean Red Ginseng on High-Fat-Diet-Induced Mouse Obesity

**DOI:** 10.3390/nu13030926

**Published:** 2021-03-12

**Authors:** Seo Yeon Lee, Hyun Gyun Yuk, Seong Gyu Ko, Sung-Gook Cho, Gi-Seong Moon

**Affiliations:** 1 Department of Preventive Medicine, College of Korean Medicine, Kyung Hee University, Seoul 02453, Korea; sylee@khut.ac.kr (S.Y.L.); epiko@khu.ac.kr (S.G.K.); 2 Department of Food Science and Technology, Korea National University of Transportation, 61 Daehak-ro, Jeungpyeong, Chungbuk 27909, Korea; hgyuk@ut.ac.kr; 3 Department of Biotechnology, Korea National University of Transportation, 61 Daehak-ro, Jeungpyeong, Chungbuk 27909, Korea

**Keywords:** gut microbiome, obesity, high-fat diet, Korean red ginseng, saponin

## Abstract

Although the anti-obesity effect of Korean red ginseng (*Panax ginseng* Meyer) has been revealed, its underlying mechanisms are not clearly understood. Here, we demonstrate an involvement of gut microbiome in the inhibitory effect of Korean red ginseng on high-fat-diet (HFD)-induced mouse obesity, and further provides information on the effects of saponin-containing red ginseng extract (SGE) and saponin-depleted red ginseng extract (GE). Mice were fed with either SGE or GE every third day for one month, and their food intakes, fat weights, plasma glucose, and insulin and leptin levels were measured. Immunofluorescence assays were conducted to measure pancreatic islet size. Stools from the mice were subjected to metagenomic analysis. Both SGE and GE attenuated HFD-induced gain of body weight, reducing HFD-induced increase of food intakes and fat weights. They also reduced HFD-increased plasma glucose, insulin, and leptin levels, decreased both fasting and postprandial glucose concentrations, and improved both insulin resistance and glucose intolerance. Immunofluorescence assays revealed that they blocked HFD-induced increase of pancreatic islet size. Our pyrosequencing of the 16S rRNA gene V3 region from stools revealed that both SGE and GE modulated HFD-altered composition of gut microbiota. Therefore, we conclude that Korean red ginseng inhibits HFD-induced obesity and diabetes by altering gut microbiome.

## 1. Introduction

Obesity, from which over 70% of the adults in the world suffer, is, in a simple interpretation, an excessive gain of body weight; but it is not simple because of its risk for diseases such as heart disease, cancer and metabolic syndrome [1,2,3,4,5]. One of comorbidities is diabetes, leading to a lowering of quality of life and life expectancy [5,6,7,8]. Diabetes is determined by high levels of blood glucose due to defects of insulin production and action, or both [8,9]. Obesity is likely to account for 80~85% of the risk of type 2 diabetes [3,8,9,10].

The human microbiome is a society of microorganisms including viruses, prokaryotes, and microbial eukaryotes in human body [6,11]. In particular, the gut microbiome is known to regulate multiple mechanisms in the body [12,13,14,15,16], thereby maintaining healthy condition [17]. Therefore, prebiotic and probiotic alterations of gut microbiome compositions are hypothesized to therapeutically reverse malfunctions, especially in terms of metabolism [18,19,20,21,22,23]. However, we still need more knowledge about the complexity of the microbiome [24]. Compositional alteration of the gut microbiome is also crucial for obesity as well as diabetes [25,26,27,28,29,30]. It has been revealed that a high-fat diet (HFD) alters the composition of the gut microbiome prior to obesity, indicating that gut microbiome derives obesity [31].

Traditional Chinese medicines (TCMs) have long been used in preventing and treating diseases in Asian countries including China, Korea (traditional Korean medicine), and Japan (Kampo medicine) [32,33]. Recent research has further highlighted TCMs as prebiotics to balance gut microbiome composition [34,35]. Among TCMs, Korean red ginseng (*Panax ginseng* Meyer), broadly used in Korea, is known to have multiple roles in the body [36]. Recent research has reported its role against obesity and diabetes [37,38,39,40,41]. Korean red ginseng is known to regulate levels of blood glucose and insulin, which is likely due to an alteration of expression levels of genes associated with metabolism [42]. Moreover, its alteration of gut microbiota was reported in obese women, although a correlation between its anti-obesity role and its alteration of microbiome was not clearly investigated [43]. However, its role in the HFD-altered gut microbiome is yet clearly explored. Moreover, while saponins are known to have an anti-obesity effect [44], there is no evidence on their regulation of the gut microbiome.

In this study, we investigated whether a role of red ginseng in preventing obesity and diabetes involves the alteration of gut microbiome composition. We further tested whether saponins are crucial for the function mentioned above if true.

## 2. Materials and Methods

### 2.1. Ginseng Extracts and Animal Experiments

Korean red ginseng extracts (saponin-containing and saponin-depleted extracts) were received from Korean Ginseng Corporation (KGC, Daejeon, Korea). Nutrient composition of saponin-containing extracts (SGE) was 20.96% of crude protein, 77.02% of carbohydrate, 0.46% of crude fat, 1.36% of crude ash, etc., and ginsenoside contents of the extracts were 2.29 mg/g of Rg1, 2.65 mg/g of Re, 6.03 mg/g of Rf, 8.55 mg/g of Rg2s, 20.71 mg/g of Rb1, 9.28 mg/g of Rc, 7.89 mg/g of Rb2, 4.1 mg/g of Rd, 2.79 mg/g of Rg3s, 5.39 mg/g of Rg3r, and 9.3 mg/g of Rh1. Nutrient composition of saponin-depleted extracts (GE) was 12.98% of crude protein, 71.39% of carbohydrate, 0.16% of crude fat, 6.73% of crude ash, etc., and the content of red ginseng polysaccharide was 108.21 mg/g. Mouse in vivo studies were authorized by Kyung Hee University Institutional Animal Care and Use Committee (KHU-IACUC, KHUASP(SE)-18-19, Kyung Hee University, Seoul, Korea). Each group contained 15 mice, which were randomly assigned. For obese groups, eight-week-old C57BL/6 male mice were fed ad libitum with 55% high-fat diet for 70 days. As a control, mice were fed ad libitum with 10% normal fat diet. To test anti-obesity effect of Korean red ginseng extracts, extracts at 235 mg/kg, which was determined by allometric scaling of 3000 mg for a human adult of 65 kg regarding maximum tolerated dose, were diluted in water and orally added two times a week for 4 weeks. Fecal pellets of each mouse were collected in sealed containers for the last 7 days of the study, and immediately stored at −80 °C with prior snap-freezing in liquid nitrogen until either fecal feeding or DNA extraction. Fecal samples were also diluted in water and orally added into the mice (*n* = 15/group). Body weight and food consumption were measured every third day. Food efficiency ratio was measured by the formula as follows: FER (%) = 100 × body weight gain (g)/food intake (g). After sacrificing mice at the end of the experiments, abdominal fat was measured and divided with body weight.

### 2.2. Tests for Blood Glucose, Insulin, and Leptin Levels

After four-hour fasting before the end of the experiments, the bloods were collected by cardiac puncture and centrifugated at 1500× *g* for 10 min. Glucose concentrations were measured with a glucometer (Accu-Check, Roche Diagnostics Co., Indianapolis, IN, USA). Insulin and leptin levels were measured by ELISA kit. (Cayman Chemical Company, Ann Arbor, MI, USA). For the glucose tolerance test, mice (*n* = 5/group) were fasted for 12 h and then 2 g/kg glucose was administered intraperitoneally. Blood samples were collected via tail vein at every 20 min for 120 min. For the insulin tolerance test, mice (*n* = 5/group) were fasted for 4 h, and then the blood samples were collected via tail vein prior to basal (time 0) as well as after intraperitoneal injection of insulin (0.75 unit/kg body weight; Merck-Millipore, Darmstadt, Germany) at 20, 40, 60, 80, 100, and 120 min. Glucose level was measured by One Touch Ultra analyzer. Insulin tolerance was confirmed by homeostatic model assessment for insulin resistance (HOMA-IR). The formula was as follows: fasting glucose level × fasting insulin level/405. Enzymatic activities of glutamic oxaloacetic transaminase (GOT) and glutamic pyruvic transaminase (GPT) were measured using commercial kits (Asanpharm, Seoul, Korea).

### 2.3. Immunofluorescence Assays

Pancreas from mice (*n* = 5/group) was paraffin-embedded and sectioned at 10 μm. Tissues were incubated with guinea pig anti-insulin (Millipore; 1:50 in dilution) or rabbit anti-glucagon (Millipore; 1:50) antibody overnight at 4 °C. After washing twice with phosphate buffered saline (PBS), tissues were incubated with tetramethylrhodamine (TRITC)-conjugated anti-guinea pig IgG (Invitrogen; 1:200) for 1 h at room temperature. For counter staining, tissues were then stained with 4’, 6-diamidino-2-phenylindole (DAPI) for 5 min. Fluorescence images were obtained under confocal microscope (LSM 800, Carl Zeiss, Oberkochen, Germany).

### 2.4. Metagenomic Study

Fecal microbiota was analyzed by a metagenomic analysis tool. The stools were obtained from mice (*n* = 15/group), saved at −80 °C, and subjected to metagenomic sequencing. Sequencing following the protocol (16S Metagenomic Sequencing Library Preparation Part #15044223 Rev. B) was conducted using Herculase II Fusion DNA Polymerase Netera XT Index Kit V2 in Ilumina platform at a commercial company (Macrogen, Seoul, Korea). To analyze the 16S rRNA gene sequences, we qualified by filtering the sequence length >200 bp, trimming the ends, filtering the number of ambiguous bases, and scoring the minimum quality. Sequences were then applied to operational taxonomic units (OTUs) at 97% identity, and selected representative sequences using the QIIME software package [45]. To compute alpha diversity (within-sample), the OTU table was rarified, and richness index was calculated based on the genera profile of groups. Then, for beta diversity (between-sample), the OTU table was used to generate weighted UniFrac distance matrix. We analyzed significant differences in the relative abundance of taxa among groups by using linear discriminant analysis effect size (LEfSe). Taxa with a value from linear discriminant analysis (LDA) of more than 2 at *p* < 0.05 were considered significantly enriched. On the basis of the relative abundance analysis using LEfSe and on the basis of the results of the Kruskal–Wallis and Wilcoxon tests, *p* < 0.05 was considered statistically significant, and the threshold for the logarithmic (LDA) score was 3.0 to 4.0. Spearman’s correlation coefficient was computed to identify correlations between anthropometric value and bacterial abundance.

### 2.5. Statistics

All biochemical experiments were performed at least three times independently for each condition. The results of multiple experiments are presented as the mean ± SEM. Statistical analyses were performed using Student’s t-tests or analysis of variance (ANOVA) followed by Tukey’s multiple comparison tests as appropriate; *p* < 0.05 was considered statistically significant. Calculations were performed using GraphPad Prism 5 (GraphPad, La Jolla, CA, USA). Box and violin plots were drawn in PlotsOfData (https://huygens.science.uva.nl/PlotsOfData/ (accessed on 11 February 2021)) [46]; the graph shows the data as dots (visibility: 0.3). A violin plot reflects the sample distribution, and an open circle indicates the median of the samples (visibility: 0.5). A vertical bar indicates for each median the 95% confidence interval determined by bootstrapping. To calculate sample size and power (alpha = 0.05, power = 80%), we applied Dunnett’s correction for the sample size adjustments to compare more than one group to a control group. A web application (https://fedematt.shinyapps.io/shinyMB (accessed on 7 March 2021)) was also applied to that analysis.

## 3. Results

### 3.1. Korean Red Ginseng Inhibits High-Fat-Diet-Induced Mouse Obesity Independently of Saponins

Korean red ginseng extract is revealed to prevent obesity and diabetes in rodent models, which is likely to due to saponins [38,39,40,44,47]. Although recent research has investigated the requirement of saponins for its function in obesity and diabetes [47,48], it is still unclear whether Korean red ginseng extract not containing saponins has the same role or not. Therefore, we first investigated both saponin-containing and saponin-depleted Korean red ginseng extract in HFD-induced mouse obesity model. 

**Figure 1:**
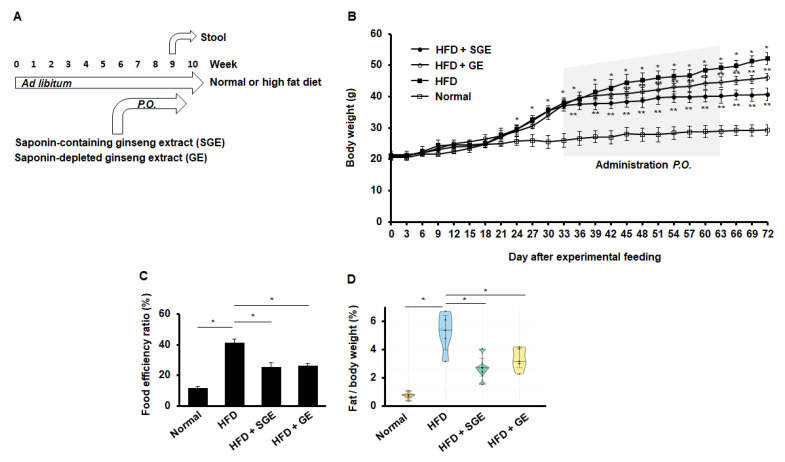
Korean red ginseng inhibits HFD-induced mouse obesity. (**A**) Experimental course. Mice (*n* = 15/group) were fed ad libitum with normal or high-fat diet for 70 days. Ginseng extract (SGE) or depleting saponins (GE) were per os added two times a week for one month. Stool samples were gathered for further research. (**B**) Body weights. Asterisks indicate statistical significances (*, *p* < 0.05; **, *p* < 0.01). A grey box indicates the administration times. Normal and HFD indicate normal and high-fat diet, respectively. (**C**) Food efficiency ratio (*n* = 15/group). *, *p* < 0.05. (**D**) A ratio of fat/body weight (*n* = 5/group). *, *p* < 0.05.

First, our test was designed to feed mice with normal and high-fat diet (HFD) or normal diet (Normal) for 70 days. Either Korean red ginseng extract (saponin-containing ginseng extract, SGE) or saponin-depleted Korean red ginseng extract (GE) was fed for 30 days during the experimental course for 70 days. Stools were gathered from mice (*n* = 15/group) for the last 7 days of the study to examine the microbiota later (Figure 1A).

When mice were fed with HFD or Normal, HFD increased body weight (Figure 1B). Importantly, when either SGE or GE was fed every third day for one month, both SGE and GE attenuated HFD-induced increase of body weight (Figure 1B). Consistently, both SGE and GE reduced the food efficiency ratio that was increased by HFD (Figure 1C). Likewise, both SGE and GE prevented HFD-induced fat accumulation (Figure 1D). Therefore, our data indicate that saponins are not critical for repressing HFD-induced increase of body weight.

### 3.2. Korean Red Ginseng Inhibits HFD-Induced Diabetic Properties Independently of Saponins

**Figure 2:**
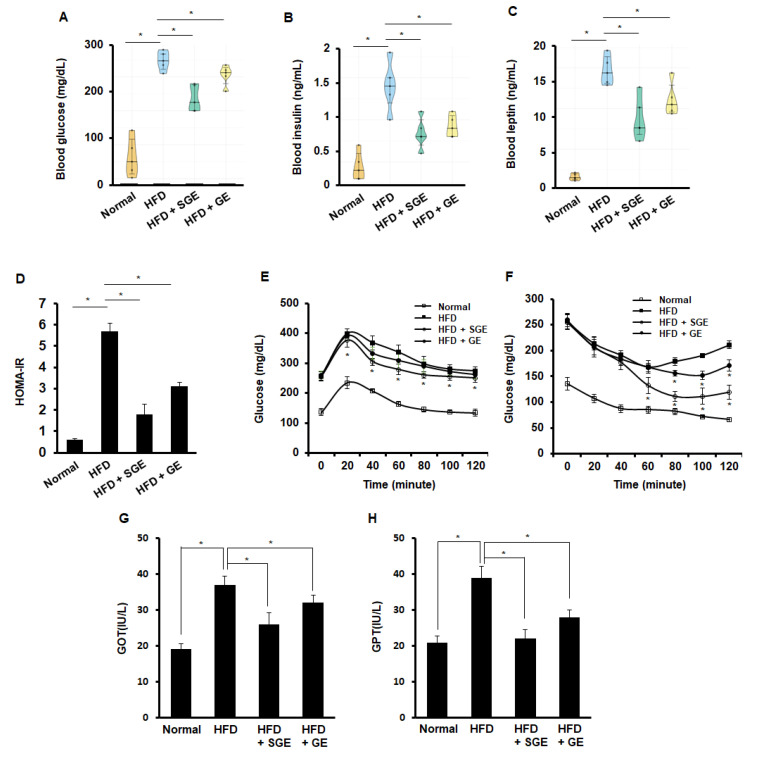
Korean red ginseng inhibits HFD-induced diabetic characteristics. (**A**–**C**) After fasting for 4 h prior to the end of the experiments, blood samples were collected (*n* = 5/group). (**A**) Blood glucose level, (**B**) blood insulin level, (**C**) blood leptin level, (**D**) homeostasis model of assessment-insulin resistance (HOMA-IR), (**E**) intraperitoneal glucose tolerance test, (**F**) intraperitoneal insulin tolerance test, (**G**) levels of glutamic oxaloacetic transaminase (GOT) activity, and (**H**) levels of glutamic pyruvic transaminase (GPT) activity. *, *p* < 0.05.

Obesity is the leading cause of diabetes. Levels of glucose, insulin, and leptin were examined from the blood of mice fed for 70 days, both SGE and GE reduced HFD-increased levels of glucose (Figure 2A), insulin (Figure 2B), and leptin (Figure 2C). Thus, SGE and GE appeared to inhibit HFD-induced diabetes, independently of saponins. When HOMA-IR was measured, both SGE and GE reduced HFD-increased HOMA-IR (Figure 2D). In the glucose tolerance test, SGE, but not GE, reduced glucose levels (Figure 2E). However, both SGE and GE reduced glucose level in insulin tolerance test, while GE was likely weaker than SGE in the effect of glucose level (Figure 2F). Consistently, both SGE and GE reduced HFD-increased GOT (Figure 2G) and GPT (Figure 2H). Therefore, our data suggest that SGE and GE may ameliorate HFD-induced characteristics of diabetes, independently of saponins.

**Figure 3:**
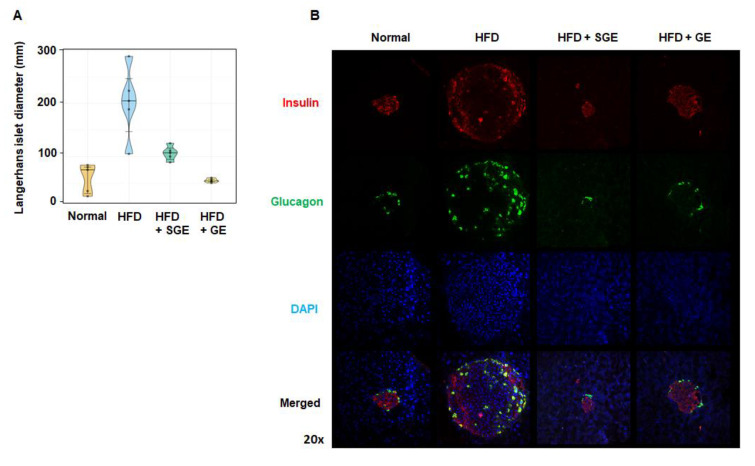
Korean red ginseng prevents HFD-induced increase of pancreatic islet size. (**A**) Langerhans islet diameter (mm) (*n* = 5/group) and (**B**) pancreatic cells expressing insulin and glucagon. Magnification 20×.

Both SGE and GE strongly blocked the HFD-induced size increase of Langerhans islets (Figure 3A), which was confirmed by examining cells releasing insulin and glucagon (Figure 3B).

### 3.3. Korean Red Ginseng Alters Gut Microbiota to Maintain Their Preventive Effects on Obesity and Diabetes Independently of Saponins

**Figure 4:**
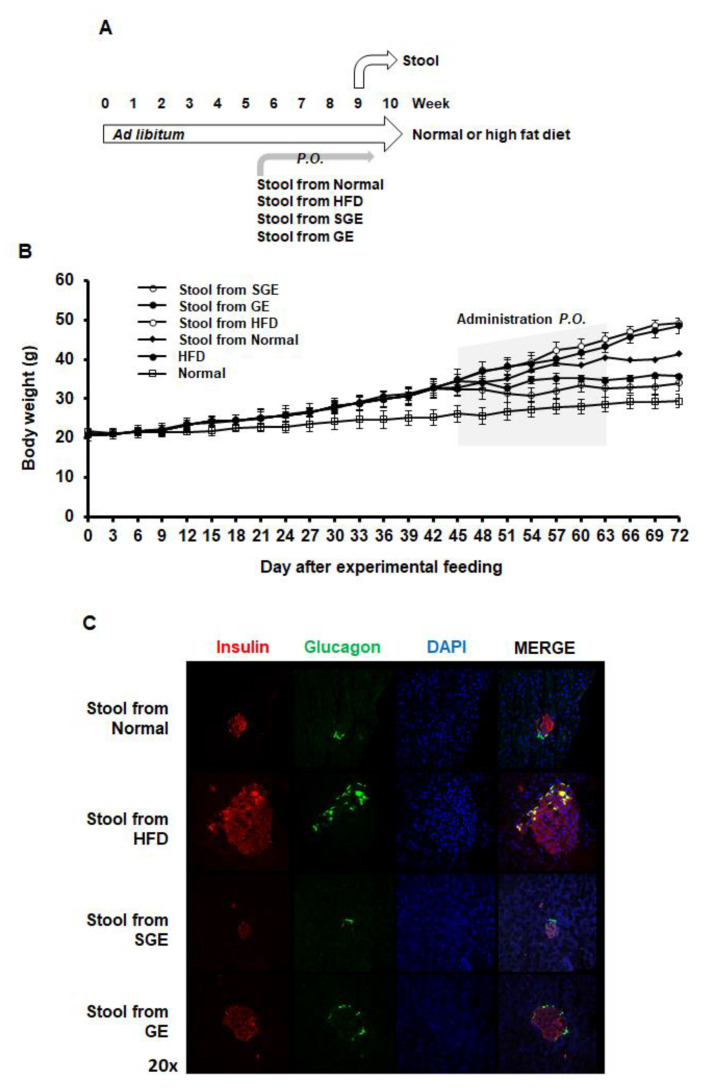
The stools from mice having Korean red ginseng extracts prevent HFD-induced obesity and diabetes. (**A**) Experimental course. Mice (*n* = 15/group) were fed ad libitum with normal or high-fat diet for 70 days. The stools from mice having the indicatives were per os added two times a week for 4 weeks. Stool samples were gathered for further research. (**B**) Gain of body weights. (**C**) Pancreatic cells expressing insulin and glucagon.

Compositional alteration of gut microbiota appears to be associated with obesity and diabetes [16,22,49]. Meanwhile, ginseng extract was reported to alter the composition of gut microbiota in obese and diabetic conditions [50]. Thus, we next examined the effects of stools from mice regarding on the effect of SGE and GE on alteration of gut microbiota composition (Figure 4A).

When the stools from mice were per os added two times a week for one month, both the stools from SGE and GE blocked HFD-induced increase body weight (Figure 4B). Accordingly, the stools from SGE and GE blocked the size increase of Langerhans islets (Figure 4C). Therefore, SGE and GE appear to reverse gut microbiota composition altered by HFD or prevent HFD-induced alteration of gut microbiota, which may prolong their preventive effect on obesity and diabetes.

Interestingly, the stool from the Normal also prevented HFD-induced increase in body weight and Langerhans islet size (Figure 4B,C), suggesting that normal microbiota composition may have a role in preventing obesity.

### 3.4. Korean Red Ginseng Extract Alters Gut Microbiota Composition

To test the effect of both SGE and GE on gut microbiota, we conducted pyrosequencing of 16S rRNA gene V3 region from the stools (Figure 1A and Figure 4A). Our metagenomic analysis showed that both SGE and GE appear to affect community richness and diversity of HFD-altered gut microbiota composition, which was determined by Chao1, Shannon, and inverse Simpson indices (Table 1).

**Figure 5:**
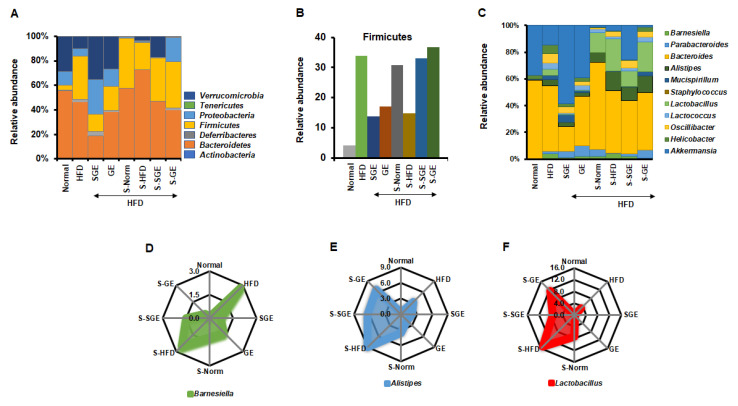
Korean red ginseng alters gut microbiota composition. (**A**) Relative abundance of gut microbiota at phylum level. (**B**) Relative abundance of Firmicutes. (**C**) Relative abundance of gut microbiota at genus level. (**D**–**F**) Relative abundance of *Barnesiella* (**D**), *Alistipes* (**E**) and *Lactobacillus* (**F**).

We next analyzed the effect of SGE and GE on gut microbiota at phylum level (Figure 5A). HFD increased Firmicutes, Deferribacteres, and Tenericutes, but reduced Proteobacteria, Verrucomicrobia, and Bacteroidetes. SGE and GE increased Verrucomicrobia and Proteobacteria reduced by HFD, and decreased Firmicutes and Tenericutes increased by HFD. Interestingly, SGE more increased Deferribacteres increased by HFD, while GE reduced it. Both SGE and GE reduced Bacteroidetes more than the HFD. Therefore, SGE and GE may have different effects on gut microbiota composition under HFD. Despite that, Proteobacteria and Verrucomicrobia appear to be important in maintaining the normal state, while Firmicutes and Tenericutes are likely crucial for obesity. It is unclear why SGE and GE reduce Bacteroidetes and show different results from Deferribacteres.

We next analyzed the composition of gut microbiota in the stool of mice fed with the HFD and the stool from the different groups of mice (Figure 5A). We found that the stool from Normal (S-Norm) increased Firmicutes, Bacteroidetes, and Actinobateria, but reduced Verrucomicrobia, Proteobacteria, and Deferribacteres, even though mice were fed with HFD (Figure 5A). We showed that the stool from Normal slightly ameliorated the gain of body weight and maintained the size of Langerhans islets (Figure 4). The stool from HFD (S-HFD) reduced Proteobacteria and Verrucomicrobia, while it increased Bacteroidetes more but reduced Firmicutes and Deferribacteres (Figure 5A). Therefore, the stools differently alter the composition of gut microbiota, which may be due to the stool itself containing dominant phyla. However, the stool from SGE (S-SGE) increased Verrucomicrobia and reduced Proteobacteria and Deferribacteres. The stool from GE (S-GE) increased Proteobacteria but reduced Verrucomicrobia with little effect on Deferribacteres (Figure 5A). Thus, while the stools from both SGE and GE prevented the gain of body weight (Figure 4A), they resulted in different effects on gut microbiota composition. 

Overall, our analysis at phylum level failed to find any representative alteration in 100% relative abundance. The ratio of Firmicutes and Bacteroidetes also did not give a unique message for a role of SGE and GE in obesity and diabetes (Normal = 0.08, HFD = 0.75, HFD + SGE = 0.75, HFD + GE = 0.51, HFD + stool from Normal = 0.71, HFD + stool from HFD = 0.30, HFD + stool from HFD + SGE = 0.74, HFD + stool from HFD + GE = 0.95). SGE and the S-SGE did not alter the ratio of Firmicutes and Bacteroidetes. However, GE and S-GE altered the ratio of Firmicutes and Bacteroidetes, while GE reduced the ratio and the stool increased it. Rather, the analysis of Firmicutes alone shows that both SGE and GE commonly reduced HFD-induced increase of Firmicutes, but the stools from Normal, SGE, and GE increased Firmicutes while S-HFD reduced it (Figure 5B). SGE and GE, therefore, are likely to alter the content of Firmicutes, which affects the status of obesity and diabetes.

We further analyzed compositional alterations of gut microbiota at genus level (Figure 5C). HFD increased *Barnesiella*, *Parabacteroides*, *Allistipes*, *Mucispirillum*, *Lactobacillus*, *Lactococcus*, *Oscillibacter*, and *Helicobacter*, but reduced *Bacteroides* and *Akkermansia*. Both SGE and GE increased *Akkermansia* reduced by HFD, further increased the *Parabacteroides* that were increased slightly by HFD, and reduced *Barnesiella*, *Bacteroides*, *Allistipes*, *Lactobacillus*, *Oscillibacter*, and *Helicobacter* increased by HFD. However, while SGE increased *Mucispirillum*, GE reduced it.

The stool from Normal increased *Bacteroides* reduced by HFD, while further reducing *Akkermansia*. It reduced *Barnesiella*, *Mucispirillum, Lactococcus*, *Oscillibacter*, and *Helicobacter* increased by HFD, while further increasing *Parabacteroides*, *Allistipes*, and *Lactobacillus*. The stool from HFD reduced *Parabacteroides* but further increased *Allistipes*. Interestingly, *Barnesiella* was not altered in the stool from HFD, while *Akkermansia* decreased. SGE reduced *Barnesiella* and increased *Akkermansia*, but GE rather increased *Barnesiella* and reduced *Akkermansia.* The stools from Normal, SGE and GE showed different effects of the balance between *Barnesiella* and *Akkermansia*.

As we failed to explain a role of SGE and GE in obesity and diabetes with 100% relative abundance, each numerical alteration was analyzed. Interestingly, HFD and S-HFD increased *Barnesiella*, which was reduced by SGE, GE, S-SGE, S-GE, and S-Norm (Figure 5D). This result strongly indicates that SGE and GE prevent obesity and diabetes via repressing HFD-induced increase of *Barnesiella.* In addition, we found that HFD-increased *Allistipes* and *Lactobacillus* were reduced by SGE and GE, and that S-SGE and S-GE reduced S-HFD-increased *Allistipes* and *Lactobacillus* (Figure 5E,F). It is plausible that S-SGE, S-GE, and S-Norm inhibit obesity and diabetes by reducing *Allistipes* and *Lactobacillus* contained in the stools that were increased by HFD. Therefore, our analysis suggests that SGE and GE have the preventive role in obesity and diabetes by regulating *Barnesiella*.

## 4. Discussion

No relevant documents were found in a Pubmed search for ‘Korean red ginseng and gut microbiome’, while recent research has revealed the association of obesity and diabetes with gut microbiome [37,38,39,51]. This study first links a role Korean red ginseng against obesity and diabetes to its compositional alteration of gut microbiota. During our research works, it was reported that GE works like SGE in a rodent type 2 diabetes model [44,48], which is supported by our present finding that SGE and GE show similar effects on phenotypes relating to obesity and diabetes.

Korean red ginseng is known to inhibit obesity by repressing HFD-induced expression of genes associated with lipid and cholesterol metabolism [51]. In this study, both SGE and GE showed similar results in obesity and diabetes although effects differed a little, suggesting that Korean red ginseng extract subtracting saponins contains compounds preventing obesity and diabetes, which is consistent with recent reports [44,48]. Importantly, our study first shows a role of Korean red ginseng in the compositional alteration of gut microbiota. In our study, Korean red ginseng repressed HFD-induced increase of Firmicutes at the level of phylum, independently of saponins. However, the stools from mice fed with Korean red ginseng or Normal did not reduced it. Rather, the stool from mice fed with HFD reduced it. Other phyla also showed no unique features associated with the inhibition of obesity and diabetes. One explanation of why Korean red ginseng extracts and the stools show differences is that bacteria from the stool may compete with gut microbiota. Another reason may be different compositions of metabolites. We still need to determine what factors really make a difference.

When we analyzed at genus level, Korean red ginseng extracts and the stools uniquely repressed HFD-induced increase of *Barnesiella* that is associated with obesity and diabetes [49,52]. In the case of *Alistipes* and *Lactobacillus* which are reported to be linked obesity and diabetes [53,54], we assume that both HFD and the stool from mice fed with HFD increase those genera. Thus, it is plausible that SGE, GE, and the stools from mice fed with SGE, GE, or Normal uniquely repress those genera under favored conditions. However, as mentioned above, what the effective factors in the stool are is still questionable.

Meanwhile, we found that SGE and GE prevented the increase of the pancreatic cells co-expressing insulin and glucagon. The cells co-expressing insulin and glucagon may be pancreatic alpha cells or progenitor cells, which are known to be increased in certain disease conditions including obesity, diabetes, and inflammation as well as in the course of their development [55,56,57,58,59,60,61,62]. Therefore, while we did not focus on this finding in the present study, it is worth studying the cells’ biological mode of action in the pancreas. Our study further showed that SGE and GE are effective in insulin resistance. However, GE failed to reduce glucose level significantly. While it is unclear whether this result is due to statistics, it is possible that GE has less effective than SGE in controlling excess glucose. In other words, SGE and GE may have different mechanisms to control obesity and diabetes. This is in line with our microbiome data. While they show similar data in the regulation of obesity and microbiome composition, detailed results are quite different between SGE and GE. Thus, we still need to determine which chemical components are responsible for this different result.

In this study, we report a role of Korean red ginseng extract in regulating the composition of gut microbiome in the prevention of obesity and diabetes, independently of saponins. However, the molecules altering gut microbiome composition remain to be deciphered.

## 5. Conclusions

Although saponins in Korean red ginseng have been considered as major contributors to its known function, this study further concluded that other components are also effective in the regulation of obesity and diabetes. While its mode of action has been focused in the host cells, this study suggests that it prolongs the effect by altering the microbiome composition. Overall, we conclude that Korean red ginseng prevents obesity and diabetes via the alteration of gut microbiome composition.

## Figures and Tables

**Table 1:** Community richness and diversity of gut microbiome.

Group	OTUs	Chao1 ^1^	Shannon ^2^	Inverse Simpson ^2^	Good Coverage
Normal	196	235.417	2.881	0.735	0.998
HFD	284	319.357	4.128	0.868	0.997
HFD + SGE	294	320.064	3.658	0.808	0.999
HFD + GE	258	298.833	4.316	0.884	0.998
HFD + stool from Normal	231	284.714	4.277	0.869	0.997
HFD + stool from HFD	301	375.391	5.065	0.928	0.998
HFD + stool from HFD + SGE	251	296.370	4.953	0.921	0.997
HFD + stool from HFD + GE	326	354.750	5.135	0.931	0.998

^1^ Chao1 was used to estimate community richness. ^2^ Shannon and inverse Simpson are diversity indices.

## Data Availability

The data presented in this study are available on request from the corresponding author.
